# Experimental and Computational Investigations of Surface Polaritons at Metal‐Oxide‐Semiconductor Interfaces

**DOI:** 10.1002/nap2.70285

**Published:** 2026-09-02

**Authors:** Humaira Zafar, Qiang Zhang, Rainer Straubinger, Ayman Rezek, Mauro Pereira, Ammar Nayfeh, Dalaver H. Anjum

**Affiliations:** ^1^ Department of Physics Khalifa University Abu Dhabi UAE; ^2^ Core Technology Platforms New York University Abu Dhabi Abu Dhabi UAE; ^3^ Department of Electrical Engineering Khalifa University Abu Dhabi UAE

## Abstract

Planar surface plasmon polaritons (SPPs) at metal–oxide–semiconductor (MOS) interfaces are central to plasmon‐assisted optoelectronic and tunneling devices, where ultrathin dielectrics govern field confinement and energy transfer. However, a direct link between electron energy‐loss spectroscopy (EELS) measurements and electromagnetic power dissipation at buried interfaces remains unclear. Here, we combine STEM–EELS with full‐wave electromagnetic simulations to investigate SPP excitation in planar $Au/SiO_{2}/Si$ and $Au/Al_{2}O_{3}/Si$ stacks with 10 nm dielectric spacers. Using a finite‐difference time‐domain approach, the fast electron is modeled as a broadband electromagnetic source, enabling decomposition of extinction into absorbed and scattered power and direct comparison with EEL spectra. We show that extinction is dominated by non‐radiative absorption, confirming that the observed resonances originate from SPPs confined to the buried Au–oxide interface. Increasing dielectric permittivity induces a redshift and modifies modal confinement, while losses remain Au‐dominated. Kramers–Kronig analysis and simulations further confirm dielectric dispersion effects and exponential evanescent decay across the oxide layer.

## Graphical abstract

STEM‐EELS and full‐wave FDTD reveal electron‐driven surface‐polariton modes at buried Au/oxide/Si interfaces with 10 nm SiO_2_ or Al_2_O_3_ spacers. Extinction is dominated by non‐radiative absorption, while oxide permittivity shifts the resonance and controls evanescent‐field penetration across the spacer into silicon.

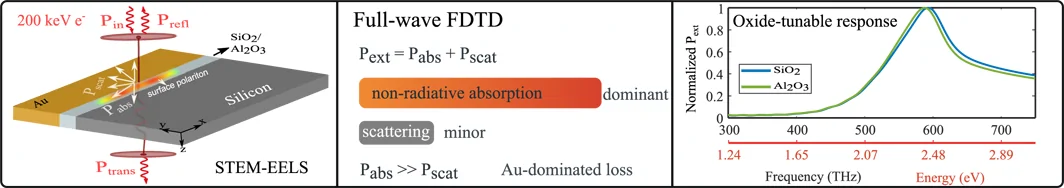

## 1 Introduction

Plasmonic nanostructures allow electromagnetic fields to be confined and controlled at dimensions well below the diffraction limit, leading to intense near‐field enhancement and tightly localized optical modes [1, 2]. These properties underpin a wide range of applications, including nanoscale sensing, spectroscopy, energy harvesting, optoelectronic devices, and the tailored engineering of light–matter interactions [3, 4]. In particular, metal‐dielectric‐semiconductor (MIS) structures have attracted significant attention due to their ability to support surface plasmon polaritons (SPPs) and gap‐plasmon modes [5, 6, 7]. The spatial confinement and decay properties of these modes are strongly influenced by the thickness of the dielectric spacer [8]. Understanding the penetration and attenuation of plasmonic fields within ultrathin dielectric layers is essential for optimizing the performance of plasmon‐assisted photodetectors, tunneling electroluminescent devices, and MIS structures [9, 10].

Electron microscopy techniques provide powerful tools for investigating plasmonic excitations at the nanometer scale [11]. Among them, electron energy‐loss spectroscopy (EELS) in a scanning transmission electron microscope (STEM) has become a highly versatile method for mapping plasmonic modes with sub‐nanometer spatial resolution. In EELS, high‐energy electrons interact with the electromagnetic response of a material, transferring part of their energy to excite plasmonic, electronic, and vibrational modes. The resulting energy‐loss spectrum reveals both the spectral features and spatial distributions of localized and propagating plasmon modes. In contrast to EELS, cathodoluminescence (CL) spectroscopy probes the radiative decay pathways of electron‐excited states by measuring photons emitted during electron‐matter interactions. By combining EELS and CL, it becomes possible to distinguish between non‐radiative (absorptive) and radiative plasmonic loss channels, yielding a comprehensive understanding of electron‐induced electromagnetic excitations [12].

While EELS and CL measurements are frequently interpreted qualitatively to map the spatial distribution and resonance energies of plasmonic modes, establishing a rigorous quantitative link to fundamental optical cross‐sections, such as absorbed, scattered, or extinct power, remains a challenge [13]. The challenge is most pronounced in the aloof configuration, where a non‐penetrating electron beam passes near a surface, enabling energy exchange primarily via evanescent near‐field electromagnetic coupling [14]. Recent research has shown that the interaction between a fast electron and a plasmonic structure can be effectively described using classical electromagnetism, with the electron modeled as a localized, broadband electromagnetic source. Extending this viewpoint, a microwave‐inspired framework has been proposed in which the electron beam is represented as a guided transverse‐magnetic (TM) mode, allowing the electron‐antenna interaction to be characterized through scattering parameters and extinction power [15]. Within this framework, the electromagnetic component of the electron energy loss is represented as an extinction process consisting of absorbed power (arising from non‐radiative plasmon damping and ohmic loss) and scattered power (corresponding to the radiative component that gives rise to cathodoluminescence).

Full‐wave electromagnetic simulations are essential for interpreting EELS and CL measurements [13]. Various numerical methods have been developed to model electron‐induced plasmon excitations, including boundary‐element, finite‐element, and finite‐difference time‐domain (FDTD) approaches [16, 17, 18]. In the FDTD framework, the electron beam is typically modeled as a sequence of phased dipoles whose temporal delays emulate the motion of a relativistic charge, enabling direct computation of wavelength‐dependent near‐field distributions, absorbed power, and radiated power [19]. This formulation further allows decomposition of the electromagnetic energy transfer from the electron into absorption and scattering components, thereby linking directly to the non‐radiative and radiative contributions of the EELS signal, respectively. In this study, we extend the electromagnetic interpretation of electron‐induced excitation to a planar metal‐oxide‐semiconductor stack. Employing electron‐beam excitation within the FDTD framework, we calculate the wavelength‐dependent electric‐field distributions, absorbed power, and scattered (cathodoluminescent) power across the visible and near‐infrared range. By fixing the electron‐beam energy and analyzing the frequency‐resolved response, we identify the plasmonic mode profiles supported by the structure and directly visualize the field decay within the ultrathin oxide layer. The resulting absorbed and scattered power spectra are combined to obtain a normalized electromagnetic extinction response, serving as a pseudo‐EELS spectrum for direct comparison with experimental EELS and CL data. This integrated simulation and analysis approach provides detailed insight into the spatial confinement, spectral behavior, and decay characteristics of plasmonic fields in metal‐oxide‐semiconductor systems, with relevance to plasmon‐enhanced electronic and optoelectronic device designs.

The literature on plasmonic nanostructures probed by STEM‐EELS is extensive, yet most experimental efforts have concentrated either on localized plasmon resonances in individual nanoparticles and nanoantennas [20, 21, 22, 23, 24], or on propagating plasmon modes in free‐standing or weakly supported metallic waveguides, including nanowires, grooves, and slot resonators [25, 26, 27]. These studies have firmly established EELS as a versatile technique for visualizing both localized and propagating plasmonic modes, enabling the observation of standing‐wave patterns, dispersion characteristics, substrate‐driven mode hybridization, and the impact of surrounding dielectrics on plasmon energies and lifetimes [28, 29]. Related studies have further demonstrated electron‐beam excitation of gap surface plasmons in metal‐insulator‐metal geometries with nanometer‐scale confinement [22], as well as electrically driven plasmon generation and modulation in tunnel junctions and active plasmonic antennas [30]. In parallel, recent advances in monochromated STEM‐EELS have enabled the exploration of low‐energy plasmons in doped semiconductors and hybrid metal‐dielectric systems, emphasizing the critical role of interfaces in determining plasmonic response [31, 32, 33]. However, a direct, systematic study of surface plasmon polaritons at a buried metal‐oxide‐semiconductor interface with practical device dimensions, like a thick gold film separated from silicon by an ultrathin Silicon dioxide layer, has not yet been systematically investigated. In particular, quantitative visualization of the spatial decay of plasmon near fields across a few‐nanometer‐thick oxide layer, which governs tunneling efficiency, non‐radiative damping, and plasmon‐semiconductor coupling in MIS architectures, has not yet been addressed within a unified electromagnetic framework [30, 34]. The present work fills this gap by combining an electromagnetic interpretation of electron beam excitation with full‐wave simulations to analyze surface plasmon polaritons supported by a planar stack of $Au-SiO_{2}/Al_{2}O_{3}-Si$ [20, 21]. By explicitly resolving the wavelength‐dependent electric field distributions, absorbed power, and scattered power under electron excitation, we directly map the plasmonic field confinement at the metal‐oxide interface and its evanescent decay into the oxide layer. This approach establishes a direct link between STEM‐EELS observables and fundamental electromagnetic quantities, providing important insights into plasmonic mode confinement, loss mechanisms, and field penetration in metal‐oxide‐semiconductor systems relevant to plasmon‐assisted electronic and optoelectronic devices.

## 2 Metal‐Oxide‐Semiconductor Structure and Electromagnetic Modeling

To quantitatively relate the experimentally measured electron energy‐loss (EEL) spectra to the electromagnetic response of the buried Au‐oxide interface, we first examine the physical layout of the STEM‐EELS experiment and its classical electromagnetic interpretation. The investigated structure consists of a 50 nm‐thick gold film deposited on a 10 nm dielectric spacer layer ($SiO_{2}$ and $Al_{2}O_{3}$), supported by a silicon substrate, as schematically illustrated in Figure 1a. Cross‐sectional TEM images of the fabricated stacks with $SiO_{2}$ and $Al_{2}O_{3}$ layers are shown in Figure 1b,c, respectively, confirming the uniformity and abruptness of the metal‐oxide interface. Because the gold layer is optically thick across the visible and near‐infrared spectra, the system isolates the surface plasmon polariton (SPP) mode at the Au‐oxide interface. The ultrathin oxide layer introduces strong field confinement and mediates the evanescent coupling between the metal and the high‐index silicon substrate, making the structure an ideal platform for studying plasmonic field penetration and decay across nanoscale dielectric gaps.

**FIGURE 1:**
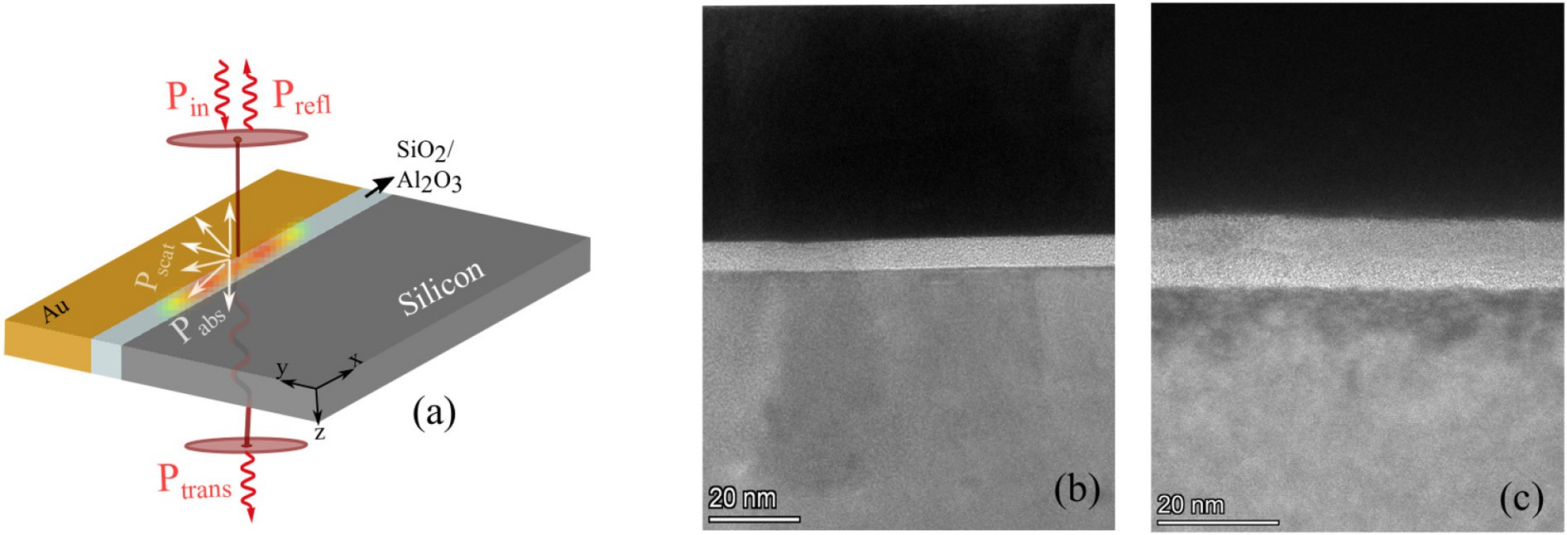
(a) Schematic of the Au–oxide–Si platform under electron‐beam excitation. The extinction of electromagnetic power carried by the electron is decomposed into scattered $\left(P_{\mathrm{scat}}\right)$ and absorbed $\left(P_{\mathrm{abs}}\right)$ contributions. Cross‐sectional TEM images of the fabricated (b) Au‐$SiO_{2}$‐Si and (c) Au‐$Al_{2}O_{3}$‐Si stacks, confirming the 10 nm oxide thickness and interface uniformity.

To probe these interface modes, we combine electron energy‐loss spectroscopy (EELS) measurements with full‐wave electromagnetic simulations under electron‐beam excitation. The total energy loss experienced by the electron due to interaction with the structure can be expressed as the time integral of the electromagnetic power dissipated:

(1) $$\Delta E=\int_{-\infty }^{\infty }P\left(t\right)\;dt$$

In the frequency domain, this energy loss is directly related to the extinction power extracted from the electron beam [13]:

(2) $$\Gamma_{\text{EEL}}\left(\omega \right)=\frac{P_{\text{ext}}\left(\omega \right)}{\pi \hbar \omega }$$

where $\Gamma_{\text{EEL}}\left(\omega \right)$ is the EEL probability density and $P_{\text{ext}}\left(\omega \right)$ represents the total electromagnetic power removed from the electron at angular frequency ω. The extinction power can be decomposed into absorption and scattering contributions,

(3) $$P_{\text{ext}}\left(\omega \right)=P_{\text{abs}}\left(\omega \right)+P_{\text{scat}}\left(\omega \right)$$

where $P_{\text{abs}}$ corresponds to non‐radiative losses (including ohmic damping of the SPP and substrate absorption), and $P_{\text{scat}}$ represents the radiative power emitted into the far field, which can contribute to cathodoluminescence. The electromagnetic interpretation of the EELS probability employed in this work follows the classical formulation developed by García de Abajo [13], in which the fast electron is treated as a relativistic point charge moving along a prescribed trajectory and interacting with the induced electromagnetic fields of the structure. In our experiments, the electron beam energy was 200 keV, corresponding to a relativistic velocity v=βc, where $\beta =\sqrt{1-{\left(1+\frac{eV}{m_{e}c^{2}}\right)}^{-2}}$. Relativistic retardation effects are therefore fully accounted for in the FDTD implementation through the time‐delayed dipole representation of the moving charge. The measurements were performed in a penetrating cross‐sectional geometry, where the electron traverses the multilayer stack and couples to interface modes via its broadband evanescent field. Under these conditions, the dominant energy‐loss channel arises from excitation of high‐momentum surface plasmon polaritons. Possible Cherenkov radiation contributions are negligible in the investigated spectral range because the electron velocity does not satisfy the Cherenkov condition v>c/n(ω) for the relevant frequencies of the oxide layers. Consequently, the measured EEL signal can be rigorously interpreted as electromagnetic extinction, decomposable into absorbed and scattered power components as described in Equations ((2), (3), (4), (5)). Overall, this formulation provides a direct electromagnetic interpretation of the EEL spectrum in terms of power dissipation and radiative channels at the buried Au‐oxide interface.

To quantitatively evaluate the extinction, absorbed, and scattered power, full‐wave simulations were performed under electron‐beam excitation conditions. In the FDTD framework, the fast electron is represented by a sequence of closely spaced dipole sources with appropriate temporal phase delays, reproducing the current density of a relativistic charge moving along a prescribed trajectory. This implementation provides broadband excitation spanning the visible and near‐infrared spectral ranges, enabling direct calculation of the induced electromagnetic fields throughout the structure. The absorbed power is obtained by volume integration of the resistive loss density:

(4) $$P_{\text{abs}}\left(\omega \right)=\frac{1}{2}\int_{V}\omega \varepsilon_{0}\varepsilon_{2}\left(\omega \right){\left|E\left(r,\omega \right)\right|}^{2}\;dV$$

where $\varepsilon_{2}$ denotes the imaginary part of the permittivity. The scattered power is determined from the net electromagnetic power flux through a closed surface A surrounding the structure:

(5) $$P_{\text{scat}}\left(\omega \right)=\oint_{A}\langle S\left(\omega \right)\rangle \cdot dA$$

where S represent the Poynting vector.

For an ideal laterally invariant multilayer, the EELS probability may alternatively be evaluated semi‐analytically using the retarded Green function of the stratified medium [13]. The semi‐analytical and FDTD approaches are based on the same classical electrodynamic description of the interaction between the moving electron and the induced field. FDTD is employed here because it provides, within a single broadband calculation, the real‐space field distributions and the absorbed and scattered powers required for the present spatial and energy‐channel analysis. The adopted EELS‐FDTD methodology has previously been benchmarked against boundary‐element calculations and experimental EELS results [19].

To analyze the spatial characteristics of the excited modes, frequency‐domain field monitors are employed to record the complex electric field distributions throughout the stack. These monitors provide wavelength‐resolved field maps, enabling direct visualization of the SPP confinement at the Au‐oxide interface and its evanescent decay into the dielectric spacer. Figure 2 depicts the electric‐field intensity profiles at 300 THz and 600 THz for both oxide stacks.

**FIGURE 2:**
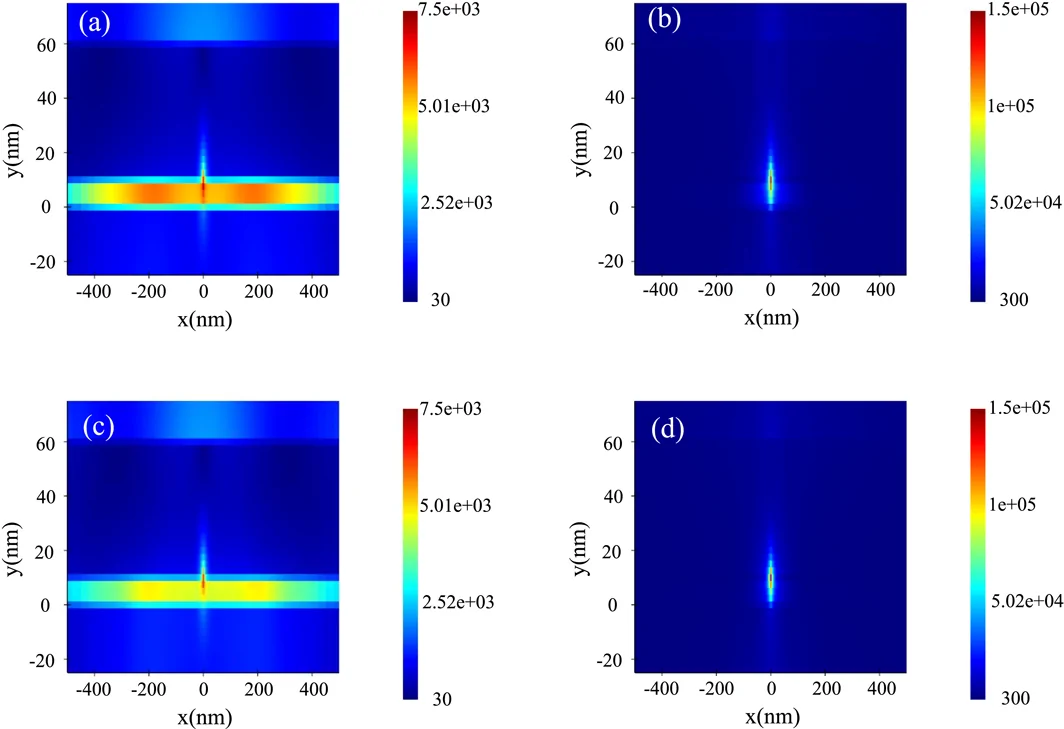
Electric field profiles under electron beam excitation at 300 THz and 600 THz, respectively for Au‐$SiO_{2}$‐Si (a, b) and Au‐$Al_{2}O_{3}$‐Si (c, d). The fields are strongly confined at the Au‐oxide interface and exhibit evanescent decay into the dielectric spacer.

The variation in electric field intensity observed between the $SiO_{2}$ and $Al_{2}O_{3}$ layers, as well as between 300 THz and 600 THz, arises from the combined effect of dielectric permittivity and frequency dependent SPP confinement. At 300 THz, Figure 2a,c depict the field distributions for Au‐$SiO_{2}$‐Si and Au‐$Al_{2}O_{3}$‐Si, respectively, exhibiting a laterally extended mode confined along the Au‐oxide interface. At this frequency, the Au‐$SiO_{2}$ interface supports stronger electron beam‐driven SPP excitation, leading to stronger induced fields and the brighter lateral lobes as depicted in Figure 2a. The higher permittivity of $Al_{2}O_{3}$ shifts the SPP resonance toward lower frequencies, as shown in Figure 2c, where the absorption peak of the $Al_{2}O_{3}$ stack appears slightly red‐shifted relative to $SiO_{2}$. As the frequency rises to 600 THz, the SPP wavevector increases in both dielectric environments, resulting in stronger modal confinement and enhanced localization of the electric field near the excitation region, as shown in Figure 2b,d. Consequently, the higher frequency generates a more confined and vertically elongated hotspot in both stacks, indicating a shorter propagation length and stronger near‐field confinement of the SPP. Compared to $SiO_{2}$, the $Al_{2}O_{3}$ case exhibits slightly stronger vertical confinement of the electric field within the oxide layer, consistent with its higher permittivity, while maintaining a similar overall spatial profile. These results demonstrate that increasing the oxide permittivity primarily modifies the confinement and resonance energy of interface modes without altering the fundamental Au‐dominated loss mechanism. Overall, the variations observed among the four profiles arise from the combined effects of dielectric‐induced SPP resonance shifts and the inherent frequency‐dependent confinement of planar surface plasmon polaritons.

The spectral decomposition of the extinction power helps to elucidate the frequency‐dependent characteristics evident in the field distributions. Figure 3a,b show the normalized absorbed and scattered power components for the Au‐$SiO_{2}$‐Si and Au‐$Al_{2}O_{3}$‐Si stacks, respectively. Across the full spectral range, extinction in both cases is primarily driven by absorption, while scattering contributes only a minor portion. The resonance peak observed in the absorbed power spectra corresponds directly to the SPP mode displayed in Figure 2. In agreement with the field maps, the Au‐$Al_{2}O_{3}$‐Si stack shows a slight redshift in the absorption peak compared to the Au‐$SiO_{2}$‐Si structure, attributed to the higher dielectric permittivity of $Al_{2}O_{3}$ and the corresponding modification of the SPP dispersion relation. As mentioned earlier, increasing the dielectric permittivity shifts the SPP resonance toward lower frequencies, since the resonance condition $\varepsilon_{m}\left(\omega \right)\approx -\varepsilon_{d}$ is satisfied at smaller ω for larger $\varepsilon_{d}$. The pronounced peak in absorbed power around the resonance frequency explains the intensified field localization seen at higher frequencies in Figure 2b,d. As the excitation frequency nears the SPP resonance, the coupling between the electron‐induced field and the interface mode becomes more efficient, resulting in enhanced energy transfer and intensified local electric field amplitudes. The strong agreement between the spectral absorption peaks and the spatial field confinement indicates that the primary contribution to the EELS signal originates from non‐radiative excitation of SPPs at the buried Au‐oxide interface.

**FIGURE 3:**
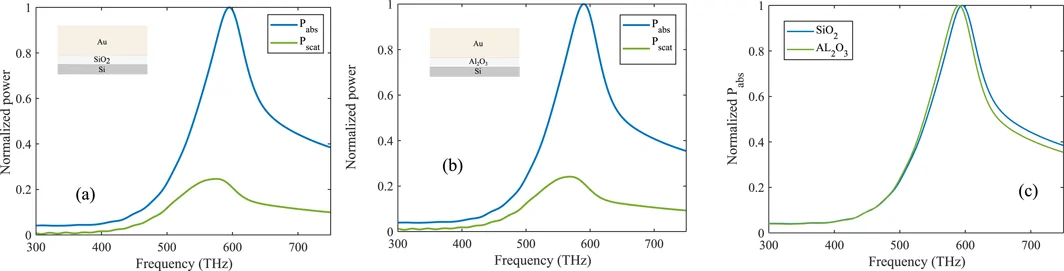
(a) Frequency‐dependent decomposition of the extinction power under electron‐beam excitation for the Au‐$SiO_{2}$‐Si stack, showing the absorbed power and scattered power, (b) corresponding extinction decomposition for the Au‐$Al_{2}O_{3}$‐Si stack. (c) Direct comparison of the normalized absorbed power spectra for $SiO_{2}$ and $Al_{2}O_{3}$ dielectric layers. In both configurations, the extinction is dominated by non‐radiative absorption, characteristic of planar surface plasmon polaritons at extended metal‐dielectric interfaces. The slight redshift of the $Al_{2}O_{3}$ resonance reflects the influence of higher dielectric permittivity on the SPP dispersion relation.

## 3 Experimental Realization and STEM–EELS Characterization

To experimentally validate the electromagnetic analysis presented in Section 1, both Au‐oxide‐Si stacks were fabricated.

### 3.1 Au‐${\text{SiO}}_{2}$‐Si Stack

A 10 nm thick ${\text{SiO}}_{2}$ thin film was deposited on silicon substrates using a plasma‐enhanced atomic layer deposition (PEALD) system. Prior to deposition, the chamber was evacuated to a base pressure below $1\times 10^{-4}$ Torr. The silicon wafer was mounted on a platen heater maintained at 200 °C throughout the deposition process. High‐purity oxygen gas (99.999%) was supplied at a flow rate of 10 sccm to a planar inductively coupled plasma (ICP) source to generate oxygen plasma. Bis(diethylamino)silane (BDEAS, Strem Chemicals Inc.) was used as the silicon precursor and introduced into the chamber through a Swagelok ALD3 valve with a pulse duration of 50 ms. The precursor was transported into the chamber by argon carrier gas (99.999% purity) at a flow rate of 5 sccm. To prevent precursor condensation, the BDEAS cylinder, ALD3 valve, gas delivery lines, and deposition chamber walls were maintained at 50 °C during deposition. The ${\text{SiO}}_{2}$ films were grown via cyclic alternating exposures of BDEAS precursor and oxygen plasma, enabling self‐limiting layer‐by‐layer growth characteristic of ALD processes. Following oxide deposition, a 50 nm‐thick gold (Au) layer was deposited by electron‐beam evaporation (PRO Line PVD 75, Kurt J. Lesker Company). Gold pellets (99.995% purity) were used as the source material. The sample stage was maintained at room temperature under a base pressure below $2.0\times 10^{-7}$ Torr. The deposition rate (1.0 Å/s) and film thickness were monitored using a quartz crystal deposition controller (GENIUS, Ferrotec GmbH).

### 3.2 Au‐${\text{Al}}_{2}O_{3}$‐Si Stack

Boron‐doped (100) p‐type silicon wafers (0.5×1.5 ${\text{cm}}^{2}$) with a resistivity of 0.001–0.005 Ω⋅cm were used as substrates. Pre‐cleaning consisted of sequential sonication in detergent, deionized water, acetone, and isopropanol (10 min each), followed by a 5 min $O_{2}$ plasma treatment. The substrates were subsequently loaded into an Okyay Tech Atomry thermal atomic layer deposition (ALD) reactor, where a 10 nm thick ${\text{Al}}_{2}O_{3}$ film was grown at 200 °C and 200 mTorr using trimethylaluminum (TMAl) and $H_{2}$O as precursors. Pulse and purge times were set to 0.25 and 45 s, respectively, resulting in a growth rate of 1.3 Å/cycle. Finally, 50 nm thick Au lines were deposited using a Moorfield MiniLab060 thermal evaporator through a shadow mask, defining metallic lines with a width of 40 μm and a spacing of approximately 500 μm.

The fabricated stacks with both oxide layers were characterized using a high‐resolution transmission electron microscope (TEM) of model Talos F200i (ThermoFisher Scientific), equipped with a cold field emission gun, a dual‐axis X‐ray energy dispersive spectrometer of solid angle 2.2 steradians, and an energy filter of model GIF Continuum 1067. The microscope was operated at the 200 kV accelerating voltage by setting it in scanning transmission electron microscopy (STEM) combined with electron energy‐loss spectroscopy (EELS) modes. Furthermore, the electron probe was made in the STEM mode by setting a semi‐convergence angle of 5.5 mrad, whereas EELS spectra were collected by setting the semi‐collection angle of 12.5 mrad. The experimental study seeks to validate the structural integrity of the multilayer platform, assess the elemental composition of the ultrathin dielectric spacer, and directly examine the plasmonic behavior at the buried metal‐oxide interface.

Figure 4 shows cross‐sectional STEM images of the fabricated Au‐oxide‐Si stacks for both dielectric spacer types. Panel (a) depicts the Au‐$SiO_{2}$‐Si structure, whereas panel (b) presents the Au‐$Al_{2}O_{3}$‐Si configuration. The high‐angle annular dark‐field (HAADF) images distinctly reveal the trilayer structure composed of the Au film, the ultrathin oxide spacer, and the silicon substrate. The oxide layer exhibits uniform thickness consistent with the nominal 10 nm design, and the interfaces are sharp with no evident interdiffusion. Elemental mapping via energy‐dispersive X‐ray spectroscopy (EDS) further verifies the compositional uniformity of the stacked structures. In the $SiO_{2}$ sample (Figure 4a), the oxygen and silicon signals are confined within the oxide layer, while the gold signal is limited to the upper metallic region. In contrast, for the $Al_{2}O_{3}$ sample (Figure 4b), the aluminum and oxygen signals are distinctly localized in the dielectric spacer, clearly separated from both the underlying silicon and the overlying gold layers. This spatial confinement of elemental distributions confirms the formation of well‐defined Au‐oxide‐Si interfaces.

**FIGURE 4:**
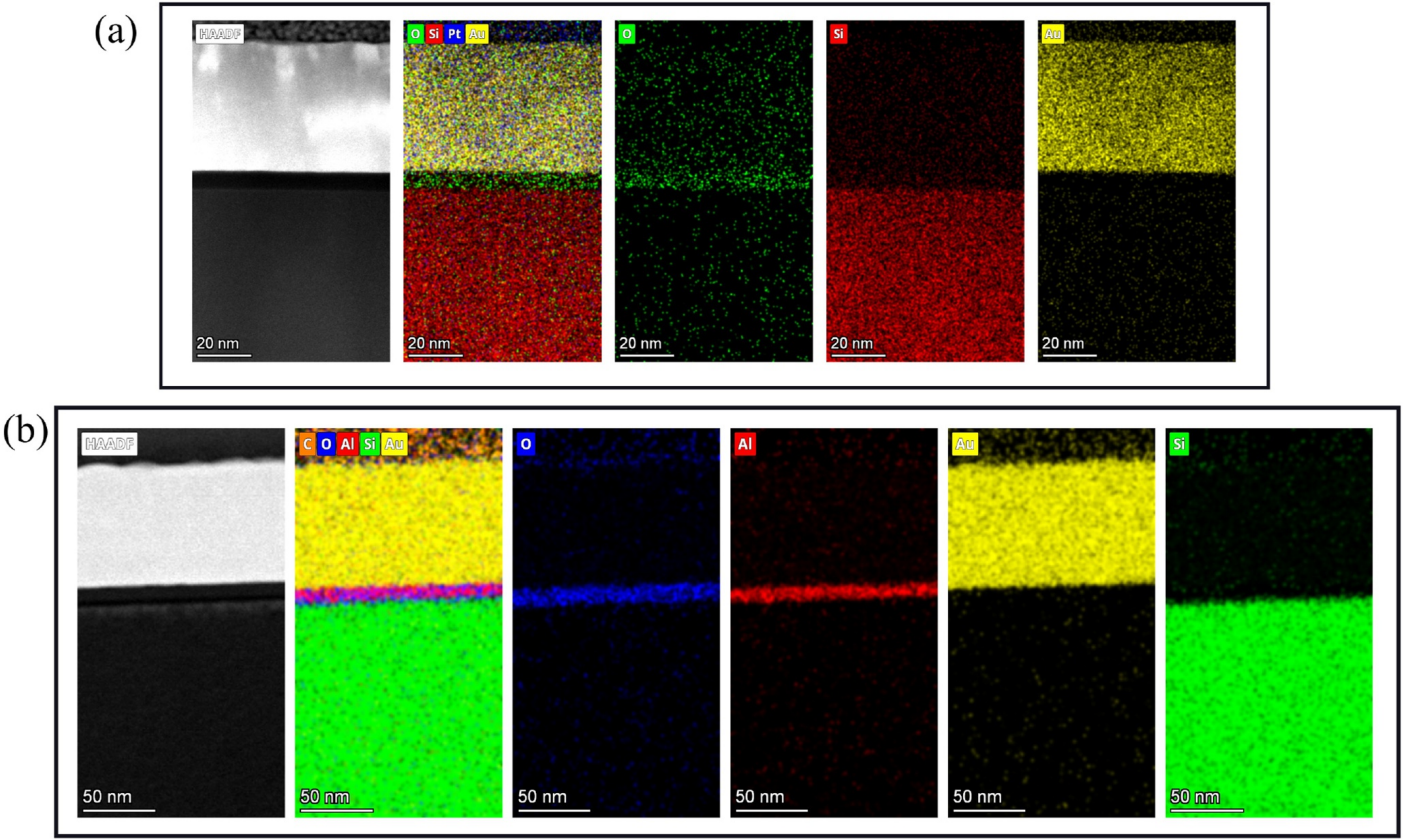
Cross‐sectional STEM characterization and elemental mapping of the fabricated Au‐oxide‐Si stacks. (a) Au‐$SiO_{2}$‐Si structure: HAADF‐STEM image (left), composite EDS elemental map, and individual elemental maps of O, Si, and Au. (b) Corresponding maps for Au‐$Al_{2}O_{3}$‐Si structure.

Figure 5 presents a comprehensive comparison between simulated electromagnetic response, experimental EELS measurements, extracted dielectric functions, and spatial field decay for the Au‐$SiO_{2}$‐Si and Au‐$Al_{2}O_{3}$‐Si stacks. Figure 5a shows the simulated normalized extinction power under electron‐beam excitation for both oxide layers, reflecting the dominance of Au intraband losses at low energies and interband transitions at higher energies. A prominent resonance appears near 580–600 THz, corresponding to excitation of the SPP at the Au‐oxide interface. The $Al_{2}O_{3}$ stack exhibits a slight redshift relative to $SiO_{2}$, in line with the higher dielectric permittivity modifying the SPP resonance condition. The experimentally acquired inelastic EELS spectra are presented in Figure 5b. Because the simulated spectra in Figure 5a and the experimental spectra in Figure 5b are expressed in different units and use independent vertical scaling, their absolute magnitudes are not directly comparable. A direct normalized comparison is presented in Figure 6.

**FIGURE 5:**
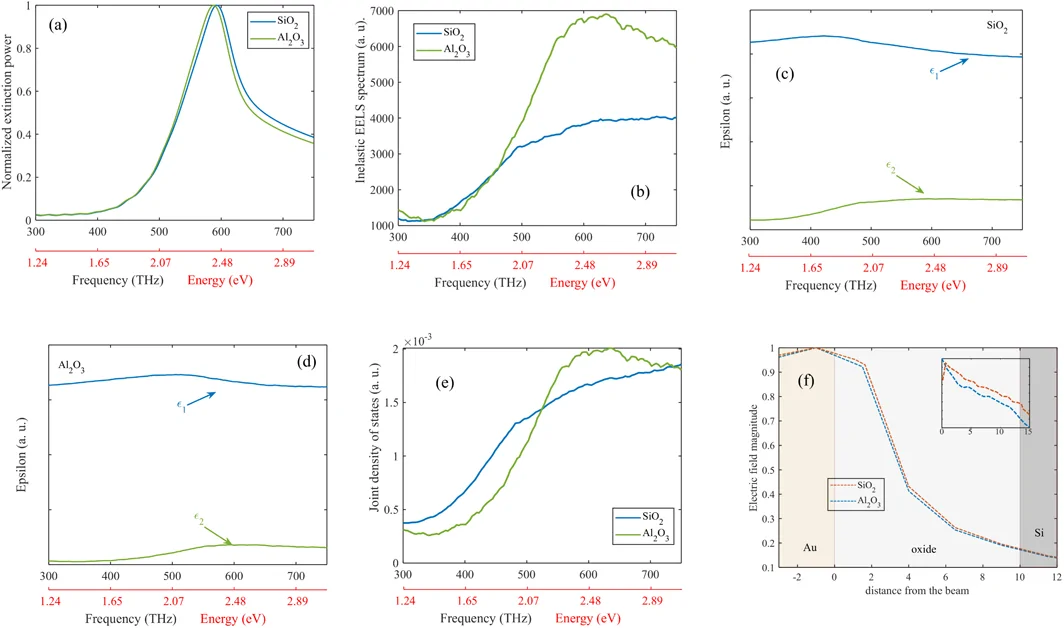
(a) Simulated normalized extinction spectra under electron‐beam excitation for Au‐$SiO_{2}$‐Si and Au‐$Al_{2}O_{3}$‐Si stacks. (b) Experimentally measured inelastic EELS spectra. (c) Real $\left(\epsilon_{1}\right)$ and imaginary $\left(\epsilon_{2}\right)$ parts of the dielectric function extracted via Kramers‐Kronig analysis for $SiO_{2}$. (d) Real $\left(\epsilon_{1}\right)$ and imaginary $\left(\epsilon_{2}\right)$ parts of the dielectric function extracted via Kramers‐Kronig analysis for $Al_{2}O_{3}$. (e) Calculated joint density of states (JDOS). (f) Simulated electric field decay across the multilayer stack at resonance; inset shows experimentally extracted decay profile. Shaded regions denote the Au, oxide, and Si layers.

**FIGURE 6:**
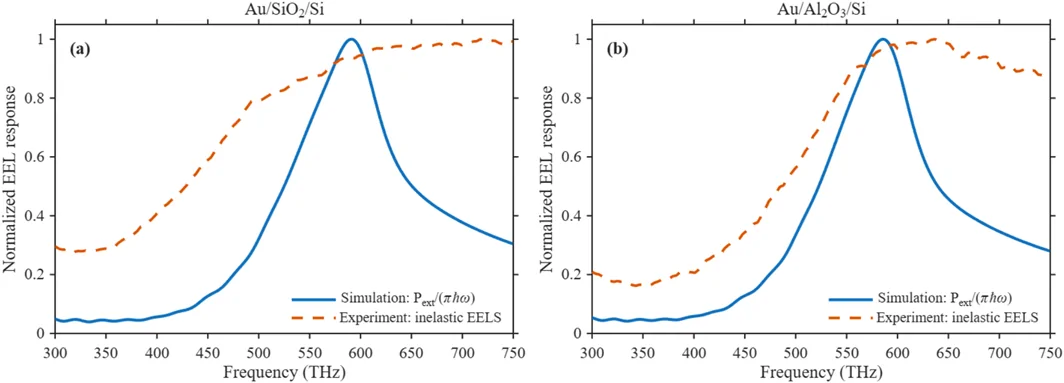
Qualitative normalized comparison between the simulated and experimental EELS responses for (a) Au‐$SiO_{2}$‐Si and (b) Au‐$Al_{2}O_{3}$‐Si. The simulated extinction power was converted to a relative EELS response according to Equation (2). The experimental curves correspond to the zero‐loss‐peak‐subtracted inelastic EELS spectra. For each material stack, the simulated and experimental curves were independently normalized to their respective maximum values over 300–750 THz range. The comparison is qualitative and is intended only to place the calculated surface‐mode response within the broad experimental low‐loss range. Quantitative agreement in absolute loss probability or complete spectral line shape is not implied.

Figure 5c,d display the real $\left(\epsilon_{1}\right)$ and imaginary $\left(\epsilon_{2}\right)$ parts of the dielectric function extracted via Kramers‐Kronig analysis for $SiO_{2}$ and $Al_{2}O_{3}$, respectively. The real part $\epsilon_{1}$ governs the SPP resonance condition through the relation $\epsilon_{m}$(ω) ≈ $-\epsilon_{d}$(ω), while the imaginary part $\epsilon_{2}$ contributes to dissipative losses and influences the resonance linewidth. The distinct dielectric dispersion profiles of $SiO_{2}$ and $Al_{2}O_{3}$ account for the observed redshift and subtle spectral differences in the extinction response. Figure 5e shows the calculated joint density of states (JDOS), providing insight into the frequency range where interband electronic transitions contribute to plasmon damping. The JDOS presented in Figure 5e corresponds to the bulk electronic structure of the dielectric spacer materials, namely $SiO_{2}$ and $Al_{2}O_{3}$, rather than to the gold layer. The JDOS was calculated using tabulated bulk dielectric functions and evaluating the interband transition contribution through the relation $J\left(\omega \right)\propto \omega \varepsilon_{2}\left(\omega \right)$, where $\varepsilon_{2}\left(\omega \right)$ is the imaginary part of the dielectric function associated with electronic transitions. This approach provides a phenomenological estimate of the available electronic states participating in optical absorption without invoking full density functional theory (DFT) calculations. Because both $SiO_{2}$ and $Al_{2}O_{3}$ are wide‐bandgap insulators, their JDOS in the investigated visible and near‐infrared spectral range primarily reflects the onset and gradual increase of higher‐energy interband transitions. The inclusion of the bulk dielectric JDOS therefore serves to contextualize the frequency‐dependent damping channels influencing the plasmon resonance, rather than to model metal interband transitions in gold. This clarification ensures that the JDOS analysis is interpreted as a bulk dielectric property relevant to loss mechanisms in the multilayer stack. Finally, Figure 5f presents the simulated electric field decay across the multilayer structure at resonance, with the inset showing the experimentally extracted decay profile. The electric field exhibits exponential attenuation within the dielectric layer, confirming the evanescent character of the SPP mode. The close agreement between simulation and experiment demonstrates that the plasmonic near field penetrates significantly across the 10 nm oxide layer before decaying in the silicon substrate. The shaded regions indicate the Au, oxide, and Si layers, emphasizing that the excitation is localized at the buried interface.

Figure 5f compares the simulated field‐decay profile with the experimentally extracted decay profile across the complete Au/oxide/Si stack. Because the oxide thickness is only 10 nm and the near field extends into the Si substrate, the decay cannot be described quantitatively using the penetration‐depth expression for an isolated metal–dielectric interface. The simulated profile was obtained directly from the full‐wave FDTD model, which includes the Au film, the finite‐thickness oxide layer, and the Si substrate. The field is concentrated near the Au–oxide boundary and decays across the oxide while remaining influenced by the underlying Si. Accordingly, we interpret the excitation as a three‐layer TM surface‐plasmon mode of the complete Au/oxide/Si stack rather than as an isolated Au/oxide‐interface SPP. This interpretation is consistent with the thickness‐dependent behavior of layered plasmonic structures [35].

It should be noted that the frequency‐dependent dielectric functions and the measured spectral response are distinct from the modal SPP dispersion, $\omega \left(k_{\|}\right)$. A direct experimental determination of the latter requires measurement of both the energy loss and the corresponding momentum transfer parallel to the interface. The present STEM‐EELS measurements were acquired in a spatially resolved configuration, and an independent momentum‐transfer coordinate was not retained. Therefore, the present measurements provide the resonance‐energy response and the transverse spatial decay across the multilayer. Still, they do not permit direct experimental extraction of the in‐plane SPP dispersion. Such a determination would require a dedicated momentum‐resolved EELS experiment.

To compare the calculated and measured spectral responses on a consistent relative scale, the simulated extinction power was converted to a relative EELS response using Equation (2). The resulting simulated spectra and the zero‐loss‐peak‐subtracted experimental inelastic EELS spectra were independently normalized to their respective maximum values over the common frequency interval of 300–750 THz, as shown in Figure 6. This normalization removes the arbitrary difference between the vertical scales used for simulation and experiment. Consequently, Figure 6 provides a qualitative normalized comparison between the calculated relative EELS response and the measured low‐loss spectra. For both stacks, the calculated surface‐mode resonance near 580–600 THz lies within the broad frequency interval over which the experimental loss response increases strongly. Nevertheless, the experimental spectra are considerably broader and retain substantial intensity at frequencies above the calculated resonance. This residual broadband response may contain contributions from multiple inelastic scattering, finite instrumental energy resolution, spatial and angular averaging within the experimental collection geometry, finite TEM‐lamella thickness, interface and layer‐thickness nonuniformities, differences between the optical properties of the fabricated films and those used in the idealized model, and additional bulk or substrate‐related excitations sampled by the penetrating electron beam. These contributions cannot be separated quantitatively using the present data and are therefore not assigned to a single loss mechanism. Accordingly, experimental intensity is not interpreted as a direct quantitative measure of modal confinement.

## 4 Conclusion

In this work, we presented a combined experimental and computational study of surface plasmon polaritons at buried metal‐oxide‐semiconductor interfaces. By integrating full‐wave electromagnetic simulations with STEM‐EELS measurements for a cold‐field‐emission gun (C‐FEG), we established a direct link between electron‐induced extinction, absorbed power, and measured energy‐loss spectra for planar Au‐$SiO_{2}$‐Si and Au‐$Al_{2}O_{3}$‐Si stacks with ultrathin dielectric spacers. The simulations reveal that electron energy loss is best understood as electromagnetic extinction, which is primarily driven by non‐radiative absorption due to planar SPP excitation at the Au–oxide interface. The resonances observed experimentally align closely with the simulated EELS profiles, demonstrating that the EEL signal is mostly attributed to non‐radiative SPP modes, rather than to radiative scattering processes. We also demonstrate that varying the permittivity of the dielectric spacer systematically alters the SPP resonance, leading to a measurable redshift and nuanced changes in modal confinement, while maintaining the underlying Au‐dominated loss mechanism. Kramers‐Kronig analysis of the EELS data reveals dielectric dispersion trends that align with the observed spectral features. Furthermore, both simulations and experiments confirm that the plasmonic near field decays exponentially across the 10 nm oxide layer. The measured decay profiles indicate that the field penetration length is on the order of the spacer thickness, highlighting a regime of strong coupling between the metal and semiconductor.

## Author Contributions

**Humaira Zafar:** methodology, writing – original draft, software, validation, investigation. **Qiang Zhang:** methodology, validation, writing – review and editing, investigation. **Rainer Straubinger:** investigation, methodology, writing – review and editing. **Ayman Rezek:** investigation, methodology, writing – review and editing. **Mauro Pereira:** investigation, methodology, writing – review and editing. **Ammar Nayfeh:** investigation, methodology, writing – review and editing. **Dalaver H. Anjum:** conceptualization, investigation, methodology, funding acquisition, writing – review and editing.

## Funding

This publication is based upon work supported by Khalifa University's Research and Innovation Grants (RIG) program under Award No. RIG‐2024‐002.

## Conflicts of Interest

The authors declare no conflicts of interest.

## Data Availability

The data used to generate the figures and tables can be obtained from the corresponding author upon reasonable request.
